# Trends and variation in the incidence of hip fracture in England before, during, and after the COVID-19 pandemic (2014–2024): a population-based observational study

**DOI:** 10.1016/j.lanepe.2025.101427

**Published:** 2025-08-13

**Authors:** James Webster, Emre Oguzman, Eva J.A. Morris, Sasha Shepperd, Xavier L. Griffin, Antony Johansen, Raphael Goldacre

**Affiliations:** a Applied Health Research Unit, Nuffield Department of Population Health, University of Oxford, UK; b Bone and Joint Health, Queen Mary University London, London, UK; c University Hospital of Wales and School of Medicine, Cardiff University, Cardiff, UK

**Keywords:** Hip fracture, Incidence, COVID-19, Epidemiology

## Abstract

**Background:**

Hip fractures are a common serious injury in older adults. The COVID-19 pandemic had a substantial impact on hip fracture prevention services, but contemporary data on incidence are scarce. We investigated recent trends and variation in the incidence of hip fracture in England.

**Methods:**

A population-based study was conducted of hip fracture hospital presentations in adults aged ≥50 years using English national secondary care data (January 2014–October 2024). Trends in incidence were compared across pre-pandemic (January 2014–February 2020), pandemic (March-2020–July-2021), and post-pandemic periods (August 2021–October 2024). Variation by age, sex, and area-level deprivation were explored.

**Findings:**

From 2014 to 2024, there were 704,762 hip fractures in 669,101 patients. Age-standardised incidence rates steadily declined from 2014 to 2019 from a mean monthly rate of 28.0–26.4 per 100,000 population. Incidence rates were below expected levels during the pandemic (IRR 0.96, 95% CI 0.94–0.97) but above expected levels in the post-pandemic period (IRR 1.03, 95% CI 1.01–1.04), resulting in 5595 more hip fractures than expected from August 2021 to October 2024. Hip fractures were more common in women, but temporal trends were consistent by sex. Incidence rates were higher in the most compared with the least deprived quintile; these inequalities remained largely unchanged by 2024 (IRR, 95% CI 1.67 [1.45–1.93] in men; 1.30 [1.19–1.42] in women).

**Interpretation:**

This study highlights an excess of hip fracture presentations to hospital since the COVID-19 pandemic, and continued disparities in incidence by deprivation, despite an overall decreasing long-term trend. More equitable prevention strategies are needed, such as through more widespread screening for fracture risk and better coverage of fracture liaison services.

**Funding:**

Authors are supported by the NIHR Oxford BRC.

**Research in context**

**Evidence before this study**

We searched PubMed for published population-based studies that reported on the incidence of hip fracture from 1 January 2010 until 13 May 2025 using the search terms “hip fracture” and “incidence”, without restrictions on language or country. Previously published studies have reported an overall reduction in hip fracture incidence from 2005 to 2018, and a higher incidence rate with increasing levels of social deprivation. However, large-scale, contemporary data that examines demographic variation and includes the COVID-19 pandemic and post-pandemic periods are lacking.

**Added value of this study**

This large-scale population-based study is one of the most extensive analyses to date on the incidence of hip fracture, and is the first to report demographic-specific rates before, during, and after the COVID-19 pandemic. Our findings extend those of prior studies by showing that following a long-term decrease in hip fracture incidence from 2014 until 2019, there has been a clinically relevant excess of hip fracture presentations to hospital since the COVID-19 pandemic. Additionally, we show that previously documented disparities in hip fracture incidence by deprivation have remained unchanged over the last decade. Future updates to these analyses will continue to track these rates via an online interactive tool to support public health messaging and policy implementation aimed at preventing and managing hip fracture.

**Implications of all the available evidence**

These findings, combined with existing literature, imply there was a decreased burden from hip fractures to healthcare systems during the pandemic, followed by an increased burden thereafter, and additionally, that more equitable hip fracture prevention strategies are needed to address unchanged disparities. These may include more widespread screening for fracture risk and better coverage of fracture liaison services, and more support to help older people stay fit and well. Continued surveillance of demographic-specific hip fracture rates and health inequalities will help to track success of prevention measures, especially in anticipation of potential future public health crises.

## Introduction

Hip fractures are the most common serious injury in older adults and are life-changing events. Patients often experience a loss of independence and health complications, and mortality in the first year after hip fracture is high, varying from 12.1% to 35.8% globally.^1^ The economic cost is estimated at 1.4% of the total healthcare burden in established market economies,^2^ or £2 billion annually to the National Health Service (NHS) in England.^3^ Improvements in life expectancy, particularly in older adults with multiple health issues, means that the number of annual cases has been projected to increase in many countries.^1^^,^^4^^,^^5^ Preventing hip fractures is therefore a public health priority.

Hip fractures disproportionately affect women and more deprived populations.^6^ Many countries have made efforts to prevent hip fractures in the community and in hospital by implementing falls prevention programs, fracture risk assessment tools alongside bone density scans, and fracture liaison services (FLS) to reduce the risk of second hip fracture by providing follow-up care, often including treatment.^7^^,^^8^ These services reduce the incidence of hip fracture and are cost-effective,^9^ but are not yet routine in many countries, and their implementation varies widely across England.^10^^,^^11^ Reports from a national hip fracture audit in England (the National Hip Fracture Database, NHFD) have shown an increase in the total number of hip fractures over the last decade.^12^ These data show an increasing overall burden of hip fracture on healthcare services, but do not account for changes in population size over time, or population ageing or demographic distributions, and so are limited for understanding temporal change or demographic variation. A reduction in hip fracture incidence, after accounting for population ageing, has been reported over the past two decades in some countries,^1^ indicating improved prevention. The most recent data in the UK has indicated a decline in rates up to 2018 using a 24% sample of the UK population^1^; hospital admissions data provides close to complete coverage of all hip fractures in England, but hip fracture rates using this data have not been reported in over a decade.^6^ Understanding how population-based incidence rates vary over time and across social and demographic characteristics is fundamental to understanding progress in prevention, and is needed to identify populations where prevention strategies should be prioritized for the largest benefits. Despite nationwide efforts to tackle health inequalities in England,^13^ it is not known if those related to hip fracture have changed over the last decade.

Evidence from several countries indicates that during the COVID-19 pandemic there was a reduction in emergency admissions for hip fractures,^14^, ^15^, ^16^, ^17^ partly due to people being at home and avoiding activities which put them at risk of falls, and partly due to the excess mortality that occurred in older populations from COVID-19 infection.^18^ Screening for high-risk fracture patients was at reduced capacity during the pandemic, and continues to have ongoing recovery challenges.^10^^,^^19^^,^^20^ For example, most services in England paused all planned DXA scans, which are used to diagnose osteoporosis and inform prophylactic treatment to prevent fractures, and as of January 2025, more than 58,000 patients were on the waiting list for a DXA scan, equivalent to around double pre-pandemic figures.^10^^,^^21^ Primary care prescriptions of anti-fracture medication also declined in 2020, but returned to pre-pandemic levels by 2021.^10^ Many adults therefore did not receive—and may continue to not receive—timely anti-fracture treatment. Similar service disruption occurred in other countries, including delays to DXA scanning, interruptions in the supply of medications, and reductions in the number of fracture risk assessments conducted.^19^^,^^22^ The impact of this on-going disruption on hip fracture rates has been reported again only in terms of total counts in England,^12^ and not across different demographic groups or in other countries. For prevention strategies to be optimally effective and equitable they need to be informed by contemporary data on the incidence of hip fracture and groups at most risk.

We aimed to investigate temporal trends in hip fracture incidence in England before, during, and after the COVID-19 pandemic using national secondary care inpatient data and to investigate the role of deprivation in hip fracture risk. Future updates to these analyses, reported via an interactive online tool,^23^ will continue to track these rates to support public health messaging and policy implementation aimed at preventing and managing hip fracture.

## Methods

### Ethical approval

Ethical approval was not required for this study as we received from NHS England pseudonymised data only.

### Study design and data sources

We followed guidelines for the reporting of studies conducted using observational routinely-collected health data (RECORD) (Supplementary eTable 1).

### Case ascertainment

In this national population-based study, we used pseudonymised patient-level data from the routinely collected Hospital Episode Statistics Admitted Patient Care (HES-APC) database for England (January 2013–October 2024).^24^ This resource contains records of every day-case and inpatient admission from all NHS hospitals in England. Clinical information is available for the principal reason for admission (the primary diagnosis) alongside secondary diagnoses, coded using International Classification of Diseases-10 (ICD-10) codes, and for procedures performed, coded using Office of Population Censuses and Surveys (OPCS) codes. Individuals were included in this study if an emergency fragility hip fracture was recorded anywhere in HES-APC from January 2014 to October 2024, and they were over the age of 50 years. Including hip fracture codes as non-primary diagnoses allowed us to capture both individuals admitted with a hip fracture and those who sustained a hip fracture in hospital. Hip fractures were identified as records where any relevant diagnosis or operation code was recorded (see Supplementary eTable 2 for code lists); this approach provides similar case ascertainment to publicly available counts from the NHFD (Supplementary eFig. 1).^12^ High-impact traumatic fractures related to transport accidents were omitted (Supplementary eTable 2). Each person could have multiple hip fractures. To avoid double-counting presentations where patients were readmitted to hospital for follow-up care, a 180-day washout period was applied between events, such that multiple presentations within 180 days were considered a single event. The washout period was based on washout periods used previously.^1^ Shorter (90-days) and longer (365 days) washout periods were applied in sensitivity analyses.

### Demographic variables

Demographic information included age, sex, and Index of Multiple deprivation (IMD) area-level deprivation.^25^ Missing data was negligible, so these records were excluded. The most amount of missing data was for IMD (<1%).

### Population denominators

To calculate population-based rates of hip fracture, we derived mid-year population estimates for England for each year from 2014 to 2024 stratified by age group (50–90+ years in 5-year intervals), sex (men, women) and IMD quintile using published population data from the Office for National Statistics (ONS) or methods for approximation where published estimates were not yet available (Supplementary methods).^26^

### Outcomes

The primary outcome was hospital presentation with hip fracture, expressed as monthly rates per 100,000 population.

### Statistical analyses

Monthly hip fracture rates were age-standardised to the 2013 European Standard Population, and were expressed per 100,000 population by sex and deprivation level (split into quintiles). Age was categorised into 5-year bands for age-standardisation, and was collapsed into broader groups for ease of reporting in age-stratified analyses (50–59, 60–69, 70–79, 80–89, and 90+ years).

We divided the study period into pre-pandemic (January 2014–February 2020), pandemic (March 2020–July 2021), and post-pandemic periods (August 2021–October 2024) based on the timing of public health restrictions imposed in England.^27^ To compare hip fracture incidence in each month during and after the pandemic with expected levels based on the pre-pandemic trend (i.e., the number of cases that would have occurred if the pre-pandemic trend had continued), a Poisson regression model accounting for over-dispersion was fitted with monthly fracture counts as the outcome, month as a discrete integer to denote the underlying pre-pandemic temporal trend, and each month from March 2020 onwards as a categorical variable. This primary model adjusted for age (in 5-year bands), sex (men, women), and seasonality using harmonic terms.^28^ The natural logarithm of annual population estimates was applied as the offset to account for changes in population size and distribution over time and pandemic periods. The underlying equation is presented in the Supplementary methods.

We repeated this method with the pandemic and post-pandemic months collapsed into pandemic and post-pandemic periods to obtain aggregated incidence rate ratios (IRRs) for these periods, and stratified by age and sex to obtain age-sex-specific relative effects of the pandemic periods. The expected mean rate during each of these periods was then calculated by dividing the observed mean rate by the relevant IRR. Similarly, for each period, the expected number of hip fractures was calculated by dividing the observed count by the relevant IRR. The observed counts minus the expected counts provided measures of absolute difference (i.e., excess or reduction) for the pandemic and post-pandemic periods. We also derived age-sex-specific estimates of excess or reduction (age split in 10-year bands), and estimates of excess or reduction stratified by IMD (split into quintiles).

To assess the association between deprivation and hip fracture incidence over time, we inserted an interaction term between IMD and calendar year as the exposure (with 2014 as the reference year), adjusted for age and seasonality. These models were stratified by sex, as an earlier study using HES showed that the effect of deprivation on hip fracture rates in England is sex-specific.^5^ Likelihood ratio tests were conducted to compare models with vs. without deprivation × time interaction terms. Statistical significance was defined as p < 0.05. All analyses were performed using Stata (v18).

### Role of the funding source

The funder had no role in study design, data collection, analysis, interpretation, or writing of the report.

## Results

**Fig. 1:**
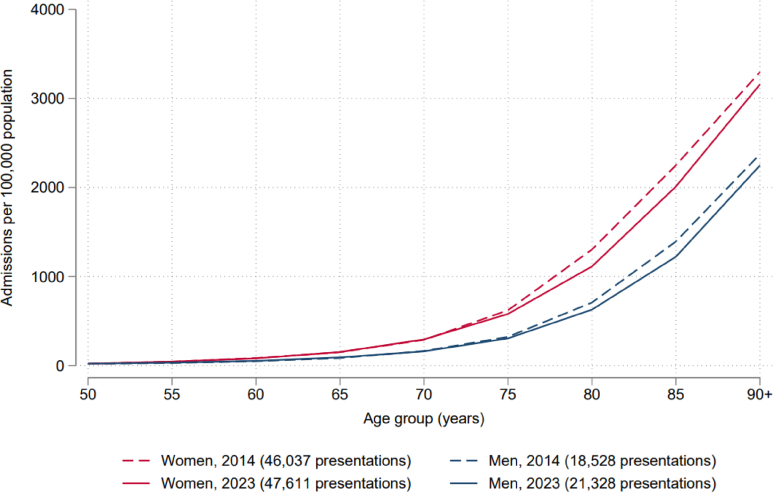
Crude rate of hip fracture presentations by age-sex-specific population in England, 2014 and 2023.

From January 2014 to October 2024, there were 704,762 hip fracture presentations to hospital among 669,101 patients (Supplementary eFig. 2). The median age at admission was 83 years (IQR 76–89 years). 69.8% of presentations occurred in women. The age-sex-distribution of hip fractures in 2014 and 2023 are presented in Fig. 1. Hip fractures were more common in women and increased exponentially with increasing age. This pattern was similar in 2020 (Supplementary eFig. 3).

### Incidence

**Fig. 2:**
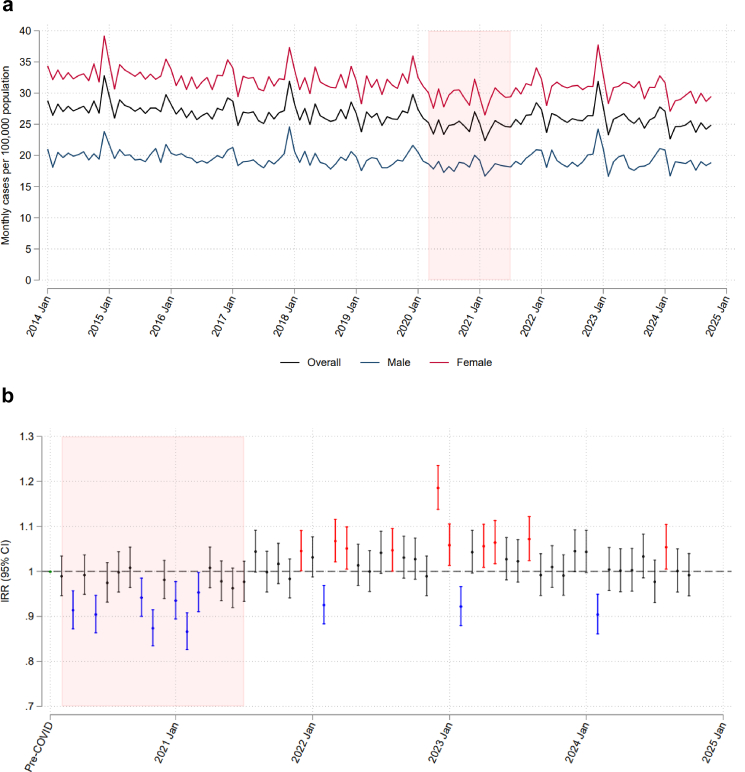
(a) Age-standardised monthly hip fracture incidence rates; and (b) relative difference between observed and expected incidence rates for each month since the COVID-19 pandemic in England, January 2014 to October 2024 in adults aged 50 years and over. Rates in part a are adjusted for age, sex, and seasonality. In part b, blue markers and confidence intervals indicate observed rates significantly below expected levels; red indicate observed rates significantly above expected levels; and black indicate no significant difference. The shaded region indicates the period of public health restrictions in England related to the COVID-19 pandemic.

Temporal trends in age-standardised monthly hip fracture rates are illustrated in Fig. 2a and b, and trends in counts and population estimates are shown in Supplementary eFig. 4. There was a slightly downward trend in incidence rates during the pre-pandemic period (IRR 0.999, 95% CI 0.999–0.999), with mean monthly rates declining from 28.0 to 26.4 cases per 100,000 population from 2014 to 2019. During the pandemic, the mean monthly rate was 24.7 per 100,000 population, which was 4% below expected levels (i.e., the counterfactual) based on the pre-pandemic trend from January 2014 to February 2020 (IRR for pandemic vs. pre-pandemic 0.96, 95% CI 0.94–0.97), resulting in 3898 (95% CI 2456–5363) fewer hip fractures than expected (Table 1). In the post-pandemic period, incidence rates were above expected levels (IRR for post vs. pre-pandemic 1.03, 95% CI 1.01–1.04), with a mean monthly rate of 25.8 per 100,000 population, resulting in an excess of 5595 (95% CI 1614–9503) hip fractures from August 2021 to October 2024. These estimates remained similar when shortening or widening the washout period for ascertaining hip fractures to 90 days or 365 days (Supplementary eTable 3), and when ascertaining hip fracture counts from the NHFD (Supplementary eTable 4).

**Table 1:** Observed hip fracture incidence in England during and after the COVID-19 pandemic, compared with expected rates based on the pre-pandemic trend, in adults aged 50 years and over.

	Pandemic (March 2020–July 2021)				Post-pandemic (August 2021–October 2024)				Overall (March 2020–October 2024)	
	Observed mean rate per 100,000	Expected mean rate per 100,000	IRR (95% CI)	Cumulative excess (95% CI)	Observed mean rate per 100,000	Expected mean rate per 100,000	IRR (95% CI)	Cumulative excess (95% CI)	Interaction by age	Interaction by sex
**Overall**	24.7	25.7	0.96 (0.94–0.97)	−3898 (−5363 to −2456)	25.8	25.0	1.03 (1.01–1.04)	5595 (1614–9503)		
**Age group**										
Men										
50–59	2.1	2.3	0.90 (0.83–0.97)	−151 (−269 to −42)	2.4	2.4	0.99 (0.91–1.09)	−19 (−347 to 281)		
60–69	5.3	5.9	0.90 (0.85–0.96)	−285 (−469 to −113)	6.0	6.0	1.00 (0.92–1.07)	5 (−508 to 484)		
70–79	18.0	18.6	0.97 (0.93–1.01)	−215 (−510 to 69)	19.3	18.2	1.06 (1.01–1.11)	990 (159–1782)		
80–89	68.2	71.0	0.96 (0.93–1.00)	−411 (−815 to −21)	71.6	68.2	1.05 (1.01–1.10)	1444 (359–2486)		
90+	189.7	186.0	1.02 (0.97–1.08)	122 (−145 to 376)	189.0	183.5	1.03 (0.97–1.10)	382 (−380 to 1099)		
Women										
50–69	2.5	2.8	0.90 (0.84–0.96)	−187 (−315 to −67)	2.8	2.8	0.99 (0.91–1.07)	−57 (−401 to 262)		
60–69	8.3	9.8	0.85 (0.81–0.90)	−748 (−1008 to −501)	9.6	9.8	0.98 (0.92–1.04)	−272 (−984 to 400)		
70–79	31.8	34.2	0.93 (0.89–0.96)	−1098 (−1610 to −602)	34.8	33.8	1.03 (0.99–1.07)	1112 (−318 to 2485)		
80–89	118.4	122.1	0.97 (0.94–0.99)	−932 (−1708 to −178)	120.5	118.1	1.02 (0.99–1.06)	1436 (−614 to 3422)	χ2 = 289.3	χ2 = 37.5
90+	259.4	259.4	1.00 (0.96–1.03)	−57 (−624 to 490)	260.1	257.5	1.01 (0.97–1.06)	382 (−1152 to 1852)	p < 0.0001	p < 0.0001

### Trends in incidence by age and sex

Temporal trends in age-sex-specific monthly hip fracture rates are illustrated in Supplementary eFig. 5a and b, and trends in age-sex-specific counts and population estimates are shown in Supplementary eFigs. 6 and 7. While pre-pandemic temporal trends in incidence were broadly consistent by age and sex, effects of the pandemic on hip fracture rates varied by age (LR test for heterogeneity χ2 = 289.3, p_interaction_ < 0.0001) and by sex (LR test χ2 = 37.5, p_interaction_ < 0.0001, Table 1). A significant dip in incidence rates below expected levels was observed in men ages 50–69 years and in women ages 50–89 years, but not in older men (70+ years) or in the oldest women (90+ years) (Table 1). The dip was most pronounced in women aged 60–69 years (IRR 0.85, 95% CI 0.81–0.90). Hip fracture rates in the post-pandemic period were generally above expected levels in older (age 70+ years) but not younger adults (Table 1).

### Trends in incidence by deprivation

**Fig. 3:**
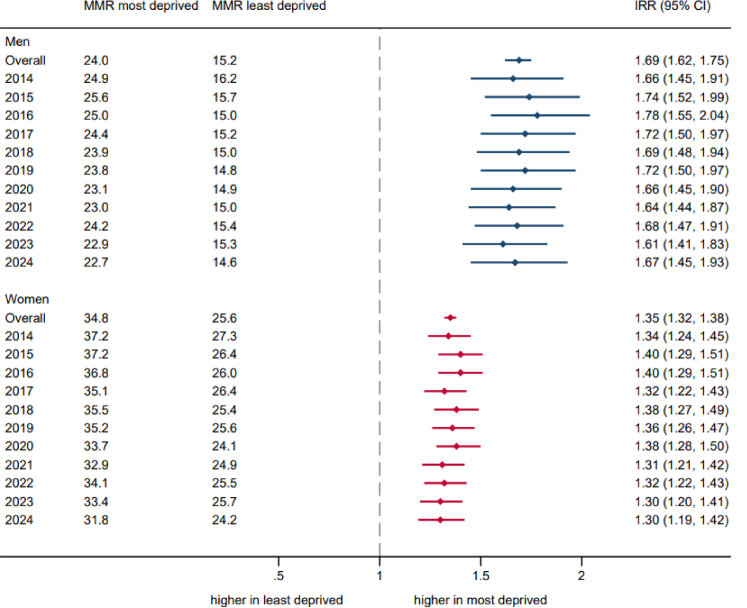
Age-adjusted hip fracture incidence rates in the most compared with least deprived populations over time by sex in adults aged 50 years and over in England, 2014 to 2024. MMR: mean monthly age-standardised incidence rate in quintiles 1 (most deprived) and 5 (least deprived) of deprivation based on the Index of Multiple Deprivation. IRR (95% CI): Incidence rate ratio (95% confidence intervals)

Age-adjusted incidence rates in the most and least deprived quintiles are shown in Fig. 3. Rates were significantly higher in the most deprived quintile in both men and women, but there was a larger deprivation gap in men (mean monthly age-standardised rate in most vs. least deprived over the study period: 24.0 vs. 15.2 per 100,000 men, and 34.8 vs. 25.6 per 100,000 women; LR test for heterogeneity by sex χ2 = 965.8, p_interaction_ < 0.0001). Although there was modest evidence of an interaction between deprivation and calendar year in women and in men (χ2 = 85.9, p_interaction_ < 0.0001 and χ2 = 58.8, p_interaction_ = 0.03), the deprivation gaps were similar in 2014 and 2024 (IRR, 95% CI for 2014 and 2024: 1.34, 1.24–1.45 vs. 1.30, 1.19–1.42 in women; 1.66, 1.45–1.91 vs. 1.67, 1.45–1.93 in men). Additionally, temporal trends in age-standardised incidence rates were broadly consistent across the most and least deprived quintiles (Supplementary eFig. 8), including during and after the pandemic (Supplementary eTable 5).

## Discussion

### Key findings

This large-scale population-based study reports recent trends in hip fracture incidence in England, and is the first to report demographic-specific rates before, during, and after the COVID-19 pandemic. Following a gradual downward trend from 2014 to 2019, there was a significant drop in hip fracture rates below expected levels in 2020 during the COVID-19 pandemic. This was followed by an increase above expected levels, resulting in an excess of 5595 more hip fractures than expected from August 2021 to October 2024. Hip fractures were more common with increasing deprivation, with a stronger relative difference in men; these inequalities remained unchanged over the last decade.

### Interpretation

The largest existing study on hip fracture incidence reported that age-standardised incidence rates decreased in many countries from 2005 to 2018.^1^ In the UK, using the Clinical Practice Research Datalink (CPRD) resource which covers 24% of the UK population, the annual hip fracture incidence rate reportedly decreased from 130.5 to 123.5 per 100,000 adults aged 50+ years from 2014 to 2018.^1^ In the current study, with close to complete coverage of the English population using national HES data, incidence rates in adults aged 50+ years also decreased, but were more than double that reported from the analysis of CPRD data. This could be due to differences between HES and CPRD in case ascertainment and the use of different age-standardisation methods. For example, Sing et al. employed the UN 2020 population, which likely resulted in lower age-standardised rates due to its younger global age structure compared to the age distribution of European populations. Additionally, while Sing et al. used diagnosis codes in isolation to capture hip fractures, we used a combination of diagnosis and operation codes to improve case ascertainment.

Data from the NHFD indicate that monthly hip fracture counts in adults aged 60+ years (including multiple admissions per person) have increased over the past decade in England.^12^ A similar trend in monthly counts was observed in this study. However, age-standardised rates decreased over the pre-pandemic period due to an increase in the ‘at-risk’ population size (i.e., the number of older adults residing in England). These data suggest that while the overall burden of hip fracture is increasing, hip fracture prevention might have been effective in reducing risk.

Prior modelling studies have projected an increase in the number of hip fractures in many countries over the decades 2020–2060,^1^ including in England,^4^ as well as an increase in hip fracture incidence rates,^5^ largely due to continued growth of the older ‘at-risk’ population. In this study, after accounting for population ageing, there was a dip in hip fracture incidence rates during the COVID-19 pandemic below expected levels. This confirms earlier findings of fewer hip fracture hospitalisations during the pandemic from a single-centre study in England,^29^ and is consistent with previous reports from population-based studies in other countries.^14^, ^15^, ^16^, ^17^^,^^30^, ^31^, ^32^

To our knowledge, no prior study has investigated trends post-pandemic. In this study, an increase in hip fracture rates above expected levels over the subsequent 41 months was observed, resulting in 5955 (3%) more cases from March 2021 to October 2024. Given that prognosis after hip fracture is poor,^23^ that hospital stays are relatively long compared to other conditions,^12^ and that treatment and management costs are high, the excess incurred following the pandemic represents an additional clinical and economic burden to health services in England. Based on current estimates of the cost of hip fractures of £14,642 per patient over 12 months from presentation,^33^ we estimate that this excess cost the NHS an additional £81.9 million.

Trends in hip fracture rates in the post-pandemic period may have been affected by several factors. Many countries, including England, experienced an excess of deaths during and after the COVID-19 pandemic, with older populations affected most,^18^ meaning many adults died before a potentially imminent hip fracture. However, in this study, the size of the older population still increased each year, albeit less than in pre-pandemic years, and hip fracture counts also increased. Older adults often require social care to conduct daily activities, but social care support in recent years has struggled to keep pace with an increasing need for care in older adults,^34^ who often have multimorbidity and are at a high risk of hip fracture. Waiting lists for osteoporosis screening and fracture risk assessment since the pandemic have grown following disruption to these services during the pandemic months, meaning many adults did not receive timely anti-fracture treatment.^10^ Osteoporosis has been globally underdiagnosed and undertreated for many years, and many patients do not receive timely anti-fracture medication^1^; these issues have been exacerbated by the pandemic; recent data in England indicate that in January 2025, 58,118 patients were waiting for a DXA scan, and one in five patients waited over six weeks, with no regions in England meeting the operational standard of less than 1% of patients waiting six weeks or more from referral.^21^ This will allow further bone deterioration and affect future hip fracture rates if screening for hip fracture risk and prescribing of anti-fracture medication do not improve, and will incur additional avoidable costs to health systems.^9^

Physical activity levels recovered to pre-pandemic levels following lifting of public health restrictions in March 2021,^35^^,^^36^ potentially leading to more falls and opportunities for hip fractures. Reduced physical activity during the pandemic may have also led to physical deconditioning and reduced mobility, particularly in those with dementia,^37^ exacerbating risk of falling when normal activity levels were resumed. Other potential reasons for the observed excess since the pandemic include an increased prevalence of risk factors for hip fracture in recent years, including more common fracture-promoting behaviours, such as increased alcohol consumption.^38^

Temporal trends in hip fracture incidence were similar across quintiles of area-level deprivation in men and in women, but it is concerning that a sustained inequality in incidence was observed. Our study updates a prior investigation using hospital inpatient data for England that reported that from 2001 to 2014, the most deprived men and women were at a 50% and 18% higher risk of hip fracture than the least deprived men and women, respectively.^6^ The deprivation gap was wider in our study, at 68% in men and 34% in women, and did not meaningfully change over the past decade. Drivers of this continued disparity may relate to the stronger hip fracture risk profile in more deprived populations, particularly men, with higher rates of alcohol consumption and smoking,^39^^,^^40^ lower physical activity levels and poorer nutrition,^41^ and a higher prevalence of comorbidities.^6^

These inequalities may also reflect potentially preventable variation in care preceding hip fractures. Prescription rates for some medications (such as denosumab) are lower in more deprived populations, and there is regional variation in DXA screening capacity and wait times, as there is currently no routine screening program for osteoporosis detection in England.^10^^,^^42^ Hip fractures commonly follow other fractures; FLSs have been established in the UK and in other countries to identify people with fractures and prevent future fractures by providing clinical assessments, treatment options, and general information to patients.^43^ While all areas of Scotland, Northern Ireland, and Wales now have an FLS, only half of all hospital trusts in England even have an FLS, and their performance against clinical standards varies widely across hospitals.^8^^,^^43^ The FLS Database 2024 annual report estimated that there were 99,220 fracture patients who did not receive care from an FLS in 2024, which is expected to lead to 596 avoidable hip fractures in 2025–2026.^11^^,^^43^ The UK Government recently pledged to achieve full coverage of FLSs across England by 2030 as part of the NHS’s long-term plan to tackle health inequalities; achieving this target is essential to preventing more hip fractures in the coming years by narrowing the inequalities highlighted in this study.^38^ If all socio-economic groups could achieve the crude incidence rate observed in the least deprived group in this study, after accounting for differences in age and sex between groups, we would expect an average of 4726 fewer hip fractures to have occurred each year over the last decade.

The findings from this study have international relevance. First, the potential drivers behind the post-pandemic increase in hip fracture rates observed in England may be generalisable internationally. These include disruption to osteoporosis screening and fracture risk assessment services since the pandemic^10^^,^^18^; physical deconditioning and reduced mobility during the pandemic^37^; and an increased prevalence of risk factors for hip fracture in recent years, including more common fracture-promoting behaviours, such as increased alcohol consumption.^38^ To our knowledge, no prior study has investigated hip fracture trends post-pandemic. Our observations may provide an indication of what has happened in other countries, and emphasise the need for other countries to provide similar data. Second, nationally representative hip fracture rates can be used for benchmarking of rates internationally to learn from the successes and failures of other countries. Our standardisation of rates in England to the European Standard Population 2013 help to facilitate these comparisons, and our continued analysis via an online dashboard will enable these comparisons to continue. Third, the deprivation gradient observed in this study highlights the need for data from other countries to determine whether social inequalities in hip fracture are consistent globally or influenced by specific social, economic, and policy contexts; such comparisons are essential to inform international efforts aimed at reducing health inequities.

### Strengths and limitations

The study was limited to patients admitted to an NHS hospital, meaning hip fractures occurring without admission could have been missed (such as those immediately leading to death). The accuracy of hip fracture case ascertainment was reliant on the accuracy of the diagnosis and operation codes used; however, hip fractures are covered by relatively few clinical codes and no reports were found of changes in coding practice during the study period. Our case ascertainment method in HES provides similar case ascertainment to the national hip fracture registry (Supplementary eFig. 1). Whilst we were able to remove hip fractures resulting from transport accidents to ascertain fragility fractures, high-energy fractures from other causes could not be distinguished or excluded. While we included individuals admitted to hospital with a hip fracture and those sustaining a hip fracture in hospital, inpatient hip fractures following elective admissions may not be captured.

The large sample size, statistical power, and representativeness achieved by using national inpatient data and official estimates of the population size for the whole of England are major strengths of this study, and enabled subgroup analyses by age, sex, and area-level deprivation. However, we relied on methods of approximation when estimating population denominators in recent years where official estimates were not yet available (see Supplementary methods for detail). Additionally, subgroup analysis by ethnicity was not possible as population denominators for ethnic groups were not available.

We were unable to report how incidence rates varied over time by occurrence setting, as this information is not recorded in the HES dataset. A study in Australia reported that hip fracture incidence dropped during the pandemic at aged care facilities and at locations away from the usual residence, but not at home or place of usual residence.^14^ Further research is needed to understand trends in incidence by setting, and could help to inform prevention strategies that account for where hip fractures occur. Finally, the use of an area-based measure of deprivation to measure health inequalities may mask absolute poverty in some cases.

### Conclusion

This study has shown a clinically relevant excess of hip fracture presentations to hospital since the COVID-19 pandemic, and continued disparities in incidence by deprivation. More equitable prevention strategies are needed to tackle the disparities observed, such as through more widespread screening for fracture risk and better coverage of fracture liaison services. Additionally, the observed excess in hip fracture rates since the pandemic emphasises an urgent need to shorten waiting lists for screening and DXA scans and ensure that prescribing and supplementation are delivered at optimal levels, particularly if the population at risk for hip fractures has increased due to behavioural changes since the pandemic. Continued surveillance of demographic-specific hip fracture rates and health inequalities is needed as health systems recover from the pandemic and as policy evolves to track the success of prevention measures, and will be provided through our interactive online tool.

## Contributors

All authors fulfil the criteria of authorship. JW conducted the literature search. JW and RG conducted the data analysis, generated the figures, and wrote the initial draft. All authors (JW, EO, EJAM, SS, XLG, AJ, and RG) contributed to study concept, design and review of the manuscript, and approved the final version.

## Data sharing statement

JW and RG had full access to all the data in the study and take responsibility for the integrity of the data and the accuracy of the data analysis. All data used in this study are available through application to NHS England at https://digital.nhs.uk/.

## Declaration of interests

JW, EO, RG and EJAM report institutional payments from NIHR Oxford Biomedical Research Centre, related to this study. XG reports funding from NIHR Barts Biomedical Research Centre, and discloses the following interests: Chair of the British Orthopaedic Trauma Society Research Committee and President Elect; Oversight Group Member for the Data Monitoring Committee for the PORTRAIT trial (NIHR159676); British Orthopaedic Association member; Fragility Fracture Liaison Service National Audit Member; BOA Research Committee Chair; Research and Innovation Committee of NHS England Outcomes and Registries Programme; Co-theme Lead for Precision Musculoskeletal Care Theme of NIHR Barts Biomedical Research Centre; and RCS England and BOA Surgical Specialty Lead (Orthopaedic Trauma) for Research.

## Appendix A Supplementary data

Supplementary eFigs. 1–8 and eTables 1–5
