# Supplementary material for: Trends and variation in the incidence of hip fracture in England before, during, and after the COVID-19 pandemic (2014–2024): a population-based observational study
Source: Lancet Reg Health Eur. 2025 Aug 13;57:101427. doi: 10.1016/j.lanepe.2025.101427 (PMC12362259; doi:10.1016/j.lanepe.2025.101427)

**Supplementary material**

**Table of contents**

[Supplementary methods 3](#_Toc202522540)

[eTable 1: Reporting of studies Conducted using Observational Routinely-collected health data (RECORD) checklist. 5](#_Toc202522541)

[eTable 2: Hip fracture definition. 16](#_Toc202522542)

[eTable 3: Observed hip fracture incidence in England during and after the COVID-19 pandemic compared with expected rates based on the pre-pandemic trend, in adults aged 50 years and over, by length of washout period. 17](#_Toc202522543)

[eTable 4: Observed hip fracture incidence in England during and after the COVID-19 pandemic compared with expected rates based on the pre-pandemic trend, in adults aged 60 years and over, based on data from the National Hip Fracture Database. 18](#_Toc202522544)

[eTable 5: Observed hip fracture incidence in England during and after the COVID-19 pandemic compared with expected rates based on the pre-pandemic trend, in adults aged 50 years and over, by deprivation, adjusted for age and seasonality. 19](#_Toc202522545)

[eFigure 1: Hip fracture presentations in the Hospital Episode Statistics Admitted Patient Care dataset and National Hip Fracture Database in adults age 60 years and older in England, January 2014 to October 2024. 20](#_Toc202522546)

[eFigure 2: Flow of hip fracture incidence from the English Hospital Episode Statistics (HES), January 2014 to October 2024, for inclusion in the analyses. 21](#_Toc202522547)

[eFigure 3: Crude rate of hip fracture presentations by age-sex-specific population in England in 2020. 22](#_Toc202522548)

[eFigure 4: Hip fracture presentations and population estimates in adults age 50 years and older in England, January 2014 to October 2024. 23](#_Toc202522549)

[eFigure 5: Age-sex-specific monthly hip fracture incidence rates in England, January 2014 to October 2024, in (a) women and (b) men aged 50 years and over. 24](#_Toc202522550)

[eFigure 6: Age-sex-specific monthly hip fracture presentations in England, January 2014 to October 2024, in (a) women and (b) men aged 50 years and older. 25](#_Toc202522551)

[eFigure 7: Age-sex-specific annual mid-year population estimates in England, January 2014 to October 2024, in (a) women and (b) men aged 50 years and older. 26](#_Toc202522552)

[eFigure 8: Monthly age-standardised hip fracture incidence rates in the most and least deprived quintiles in (a) men and (b) women aged 50 years and older in England, January 2014 to October 2024. 27](#_Toc202522553)

# Supplementary methods

*Population denominators*

We derived denominators for population-based rates using published mid-year (30 June) estimates of the usual resident population in England from the Office for National Statistics (ONS). Two sets of mid-year estimates were obtained: national rebased (i.e., post-Census 2021 revised) estimates between 2014 and 2023,^1^ and Lower layer Super Output Area (LSOA) estimates between 2014 and 2020.^2^ We used national rebased estimates to derive denominators by age group and sex, in addition to the total cohort. Estimates from 2023 were used for the 2024 data of these groupings.

English indices of deprivation measure relative deprivation in LSOAs. The latest version of the IMD (2019) is based on LSOAs from the 2011 Census,^3^ and the 2020 LSOA dataset is the last set of estimates to use the geographic boundaries defined by the 2011 Census.^2^ We derived denominators for deprivation by first mapping each IMD quintile to LSOA estimates between 2014 and 2020. LSOA estimates from 2014 to 2016 were mapped to the 2015 edition of the IMD, while estimates from 2017 to 2020 were mapped to the 2019 edition of the IMD.^3^ We then derived the yearly IMD distribution from LSOA population estimates (2014-2020), and applied this distribution to the national rebased estimates by age and sex between 2014 and 2023. The IMD distribution for the year 2020 was applied for the years 2021–2023. Estimates from 2023 were again used to approximate population estimates in 2024.

*References*

1. Office for National Statistics. *Data from: Population estimates - local authority based by single year of age.* 2023 (accessed 30 Mar 2025).
2. Office for national Statistics. *D*a*ta from: Lower layer Super Output Area population estimates (supporting information)*. 2024 (accessed 30 Mar 2025).
3. Ministry of Housing CLGt. *English indicies of deprivation 2019*. 2019 (accessed 30 Mar 2025). In: <https://www.gov.uk/government/statistics/english-indices-of-deprivation-2019>

*Protype equation for Poisson model*

The prototype equation for the Poisson regression model used to assess the effect of the pandemic periods on hip fracture rates is as follows:

log(λᵢ) = β₀ + β₁ageᵢ + β₂sexᵢ + β₃timeᵢ + β₄pandemicᵢ + sin + cos + log(popᵢ)

Where:

- λᵢ: indicates hip fracture rates at month i
- Age: dummy variable with categories 1 (50-54 years), 2 (55-59 years), 3 (60-64 years), 4 (65-69 years), 5 (70-74 years), 6 (75-79 years), 7 (80-84 years), 8 (85-89 years), and 9 (90+ years)
- Sex: binary variable 1 (male) or 2 (female)
- Time: time elapsed since January 2014 in months
- Pandemic: dummy variable with categories 0 (pre-pandemic covering January 2014 to February 2020), 1 (pandemic period covering March 2020 to July 2021), and 2 (post-pandemic covering August 2021 to October 2024).
- Sin and cos: harmonic terms to account for seasonality
- Pop: population count

# eTable 1: Reporting of studies Conducted using Observational Routinely-collected health data (RECORD) checklist.

#

|  | **Item No.** | **STROBE items** | **Location in manuscript where items are reported** | **RECORD items** | **Location in manuscript where items are reported** |
| --- | --- | --- | --- | --- | --- |
| **Title and abstract** | | | | | |
|  | 1 | (a) Indicate the study’s design with a commonly used term in the title or the abstract (b) Provide in the abstract an informative and balanced summary of what was done and what was found | Pages 1-2 | RECORD 1.1: The type of data used should be specified in the title or abstract. When possible, the name of the databases used should be included.  RECORD 1.2: If applicable, the geographic region and timeframe within which the study took place should be reported in the title or abstract.  RECORD 1.3: If linkage between databases was conducted for the study, this should be clearly stated in the title or abstract. | Pages 1-2 |
| **Introduction** | | | | | |
| Background rationale | 2 | Explain the scientific background and rationale for the investigation being reported | Pages 5-6 |  |  |
| Objectives | 3 | State specific objectives, including any prespecified hypotheses | Page 7 |  |  |
| **Methods** | | | | | |
| Study Design | 4 | Present key elements of study design early in the paper | Page 7 |  |  |
| Setting | 5 | Describe the setting, locations, and relevant dates, including periods of recruitment, exposure, follow-up, and data collection | Page 7-8 (case ascertainment) |  |  |
| Participants | 6 | *(a) Cohort study* - Give the eligibility criteria, and the sources and methods of selection of participants. Describe methods of follow-up  *Case-control study* - Give the eligibility criteria, and the sources and methods of case ascertainment and control selection. Give the rationale for the choice of cases and controls  *Cross-sectional study* - Give the eligibility criteria, and the sources and methods of selection of participants  *(b) Cohort study* - For matched studies, give matching criteria and number of exposed and unexposed  *Case-control study* - For matched studies, give matching criteria and the number of controls per case | Page 7-8 (case ascertainment)  Supplementary methods (page 3 and page 16) | RECORD 6.1: The methods of study population selection (such as codes or algorithms used to identify subjects) should be listed in detail. If this is not possible, an explanation should be provided.  RECORD 6.2: Any validation studies of the codes or algorithms used to select the population should be referenced. If validation was conducted for this study and not published elsewhere, detailed methods and results should be provided.  RECORD 6.3: If the study involved linkage of databases, consider use of a flow diagram or other graphical display to demonstrate the data linkage process, including the number of individuals with linked data at each stage. | Page 7-8 (case ascertainment)  Supplementary methods (page 3 and page 16) |
| Variables | 7 | Clearly define all outcomes, exposures, predictors, potential confounders, and effect modifiers. Give diagnostic criteria, if applicable. | Page 7-8 | RECORD 7.1: A complete list of codes and algorithms used to classify exposures, outcomes, confounders, and effect modifiers should be provided. If these cannot be reported, an explanation should be provided. | Supplementary material (page 16) |
| Data sources/ measurement | 8 | For each variable of interest, give sources of data and details of methods of assessment (measurement).  Describe comparability of assessment methods if there is more than one group | Page 7-8 (case ascertainment) |  |  |
| Bias | 9 | Describe any efforts to address potential sources of bias | Page 7-8 (case ascertainment) |  |  |
| Study size | 10 | Explain how the study size was arrived at | Page 7-8 (case ascertainment and demographic variables)  Supplementary material (page 21) |  |  |
| Quantitative variables | 11 | Explain how quantitative variables were handled in the analyses. If applicable, describe which groupings were chosen, and why | Page 7-9 (outcomes and statistical analyses) |  |  |
| Statistical methods | 12 | (a) Describe all statistical methods, including those used to control for confounding  (b) Describe any methods used to examine subgroups and interactions  (c) Explain how missing data were addressed  (d) *Cohort study* - If applicable, explain how loss to follow-up was addressed  *Case-control study* - If applicable, explain how matching of cases and controls was addressed  *Cross-sectional study* - If applicable, describe analytical methods taking account of sampling strategy  (e) Describe any sensitivity analyses | Pages 8-9 (statistical analyses) |  |  |
| Data access and cleaning methods |  | .. |  | RECORD 12.1: Authors should describe the extent to which the investigators had access to the database population used to create the study population.  RECORD 12.2: Authors should provide information on the data cleaning methods used in the study. | Pages 7-8 (ethical approval and case ascertainment)  Supplementary material (page 21) |
| Linkage |  | .. |  | RECORD 12.3: State whether the study included person-level, institutional-level, or other data linkage across two or more databases. The methods of linkage and methods of linkage quality evaluation should be provided. | Page 7-8 (case ascertainment) |
| **Results** | | | | | |
| Participants | 13 | (a) Report the numbers of individuals at each stage of the study (*e.g.*, numbers potentially eligible, examined for eligibility, confirmed eligible, included in the study, completing follow-up, and analysed)  (b) Give reasons for non-participation at each stage.  (c) Consider use of a flow diagram | Supplementary material (page 21) | RECORD 13.1: Describe in detail the selection of the persons included in the study (*i.e.,* study population selection) including filtering based on data quality, data availability and linkage. The selection of included persons can be described in the text and/or by means of the study flow diagram. | Supplementary material (page 21) |
| Descriptive data | 14 | (a) Give characteristics of study participants (*e.g.*, demographic, clinical, social) and information on exposures and potential confounders  (b) Indicate the number of participants with missing data for each variable of interest  (c) *Cohort study* - summarise follow-up time (*e.g.*, average and total amount) | Page 10  Supplementary material (page 21)  Figure 1 |  |  |
| Outcome data | 15 | *Cohort study* - Report numbers of outcome events or summary measures over time  *Case-control study* - Report numbers in each exposure category, or summary measures of exposure  *Cross-sectional study* - Report numbers of outcome events or summary measures | Page 10  Figure 2a |  |  |
| Main results | 16 | (a) Give unadjusted estimates and, if applicable, confounder-adjusted estimates and their precision (e.g., 95% confidence interval). Make clear which confounders were adjusted for and why they were included  (b) Report category boundaries when continuous variables were categorized  (c) If relevant, consider translating estimates of relative risk into absolute risk for a meaningful time period | Pages 10-11  Figures 2a and b  Figure 3  Table 1 |  |  |
| Other analyses | 17 | Report other analyses done—e.g., analyses of subgroups and interactions, and sensitivity analyses | Pages 10-11  Table 1  Supplementary material |  |  |
| **Discussion** | | | | | |
| Key results | 18 | Summarise key results with reference to study objectives | Page 11 |  |  |
| Limitations | 19 | Discuss limitations of the study, taking into account sources of potential bias or imprecision. Discuss both direction and magnitude of any potential bias | Page 15-16 | RECORD 19.1: Discuss the implications of using data that were not created or collected to answer the specific research question(s). Include discussion of misclassification bias, unmeasured confounding, missing data, and changing eligibility over time, as they pertain to the study being reported. | Page 14 |
| Interpretation | 20 | Give a cautious overall interpretation of results considering objectives, limitations, multiplicity of analyses, results from similar studies, and other relevant evidence | Pages 11-15 |  |  |
| Generalisability | 21 | Discuss the generalisability (external validity) of the study results | Page 15 |  |  |
| **Other Information** | | | | | |
| Funding | 22 | Give the source of funding and the role of the funders for the present study and, if applicable, for the original study on which the present article is based | Page 9 (role of the funding source)  Page 17 (acknowledgements) |  |  |
| Accessibility of protocol, raw data, and programming code |  | .. |  | RECORD 22.1: Authors should provide information on how to access any supplemental information such as the study protocol, raw data, or programming code. | Page 17 |

#

# eTable 2: Hip fracture definition.

| **Hip fracture diagnosis (ICD-10)** | |
| --- | --- |
| S720 | Fracture of neck of femur |
| S721 | Pertrochanteric fracture |
| S722 | Subtrochanteric fracture |
| S729 | Fracture of femur, part unspecified |
| OR | |
| **Hip fracture operation (OPCS-4)** | |
| W241 | Closed reduction of intracapsular fracture of neck of femur and fixation using nail or screw |
| W461 | Primary prosthetic replacement of head of femur using cement |
| W471 | Primary prosthetic replacement of head of femur not using cement |
| W191 | Primary open reduction of fracture of neck of femur and open fixation using pin and plate |
| W481 | Primary prosthetic replacement of head of femur NEC |
| NOT | |
| **Motor vehicle accident (ICD-10)** | |
| V01-V99 | V01-V99 Transport accidents |

# eTable 3: Observed hip fracture incidence in England during and after the COVID-19 pandemic compared with expected rates based on the pre-pandemic trend, in adults aged 50 years and over, by length of washout period.

| **Pandemic period** | **Observed mean monthly rate** | **Expected mean monthly rate** | **IRR (95% CI) vs pre-pandemic trend** | **Cumulative excess (95% CI)** |
| --- | --- | --- | --- | --- |
| **90-day washout** |  |  |  |  |
| Pre-pandemic (January 2014 - March 2020) | 27.4 |  | Reference |  |
| Pandemic (March 2020 - July 2021) | 24.9 | 25.9 | 0.96 (0.94 to 0.97) | -4086 (-5607 to -2589) |
| Post-pandemic (August 2021 - October 2024) | 26.0 | 25.5 | 1.02 (1.01 to 1.05) | 5920 (1817 to 9949) |
| **365 day washout** |  |  |  |  |
| Pre-pandemic (January 2014 - March 2020) | 26.8 |  | Reference |  |
| Pandemic (March 2020 - July 2021) | 24.4 | 25.4 | 0.96 (0.94 to 0.97) | -3787 (-5240 to -2355) |
| Post-pandemic (August 2021 - October 2024) | 25.6 | 24.9 | 1.03 (1.01 to 1.05) | 5769 (1818 to 9647) |

90-day (and 365-day) washout periods indicate that hip fractures occurring within 90 days (or 365 days) are considered a single event.

# eTable 4: Observed hip fracture incidence in England during and after the COVID-19 pandemic compared with expected rates based on the pre-pandemic trend, in adults aged 60 years and over, based on data from the National Hip Fracture Database.

| **Pandemic period** | **Observed counts** | **Observed mean monthly rate** | **Expected mean monthly rate** | **IRR (95% CI) vs pre-pandemic trend** | **Cumulative excess (95% CI)** |
| --- | --- | --- | --- | --- | --- |
| Pre-pandemic (January 2014 - March 2020) | 371386 | 24.9 | - | Reference |  |
| Pandemic (March 2020 - July 2021) | 83265 | 23.0 | 24.2 | 0.95 (0.92 to 0.98) | -4349 (-6999 to -1777) |
| Post-pandemic (August 2021 - October 2024) | 222564 | 24.9 | 23.9 | 1.04 (1.00 to 1.07) | 8061 (452 to 15,408) |

#

# eTable 5: Observed hip fracture incidence in England during and after the COVID-19 pandemic compared with expected rates based on the pre-pandemic trend, in adults aged 50 years and over, by deprivation, adjusted for age and seasonality.

|  | **Pre-pandemic (January 2014–February 2020) trend, IRR (95% CI)** | **Pandemic (March 2020–July 2021) vs counterfactual, IRR (95% CI)** | | **Post-pandemic (August 2021–October 2024) vs counterfactual, IRR (95% CI)** | |
| --- | --- | --- | --- | --- | --- |
|  |  | **IRR (95% CI)** | **Cumulative excess (95% CI)** | **IRR (95% CI)** | **Cumulative excess (95% CI)** |
| **IMD** |  |  |  |  |  |
| 1 (most deprived) | 0·999 (0·999 to 0·999) | 0·96 (0·93 to 0·98) | -612 (-1106 to -300) | 1·01 (0·98 to 1·04) | 354 (-730 to 1377) |
| 2 | 0·999 (0·999 to 0·999) | 0·96 (0·94 to 0·99) | -639 (-978 to -155) | 1·02 (0·99 to 1·05) | 737 (-380 to 1789) |
| 3 | 0·999 (0·999 to 0·999) | 0·96 (0·93 to 0·98) | -700 (-1265 to -343) | 1·05 (1·02 to 1·08) | 2025 (834 to 3151) |
| 4 | 0·999 (0·999 to 1.000) | 0·95 (0·93 to 0·97) | -923 (-1319 to -542) | 1·02 (0·99 to 1·05) | 868 (-447 to 2109) |
| 5 (least deprived) | 0·999 (0·999 to 0·999) | 0·98 (0·95 to 1·00) | -353 (-910 to 0) | 1·05 (1·02 to 1·08) | 2054 (846 to 3194) |

IMD: Index of multiple deprivation. IRR (95% CI): incidence rate ratio (95% confidence intervals).

# eFigure 1: Hip fracture presentations in the Hospital Episode Statistics Admitted Patient Care dataset and National Hip Fracture Database in adults age 60 years and older in England, January 2014 to October 2024.


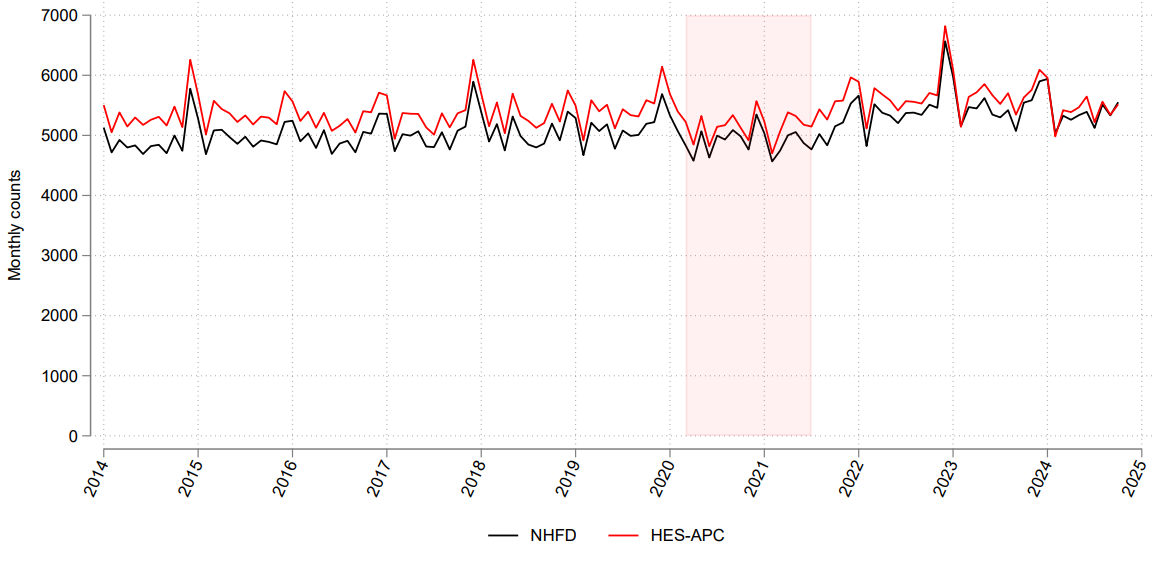


Hip fracture counts for England from the National Hip Fracture Database (NHFD) were obtained from the NHFD’s website (<https://www.nhfd.co.uk/20/NHFDcharts.nsf/vwCharts/OverallPerformance>). Hip fracture counts from the Hospital Episode Statistics Admitted Patient Care dataset were derived using ICD codes detailed in eTable 2, with a 180-day washout period applied (no restrictions were applied regarding the cause of the hip fracture).

# eFigure 2: Flow of hip fracture incidence from the English Hospital Episode Statistics (HES), January 2014 to October 2024, for inclusion in the analyses.


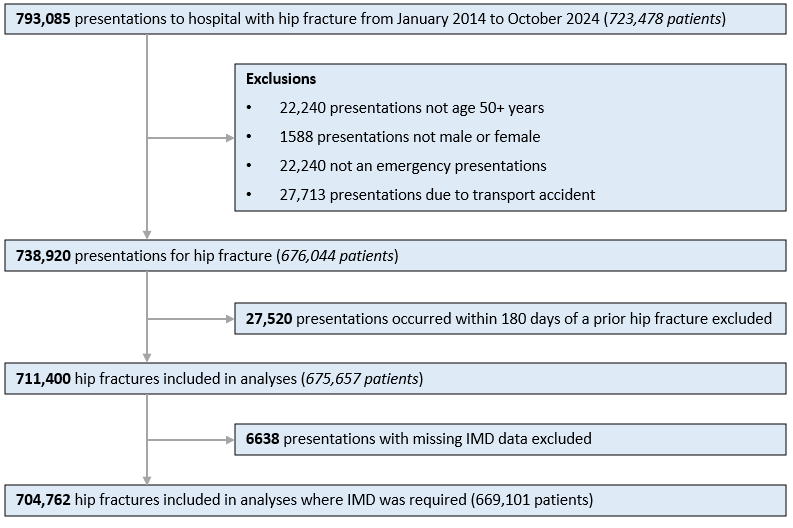
IMD: Index of multiple deprivation.

# eFigure 3: Crude rate of hip fracture presentations by age-sex-specific population in England in 2020.


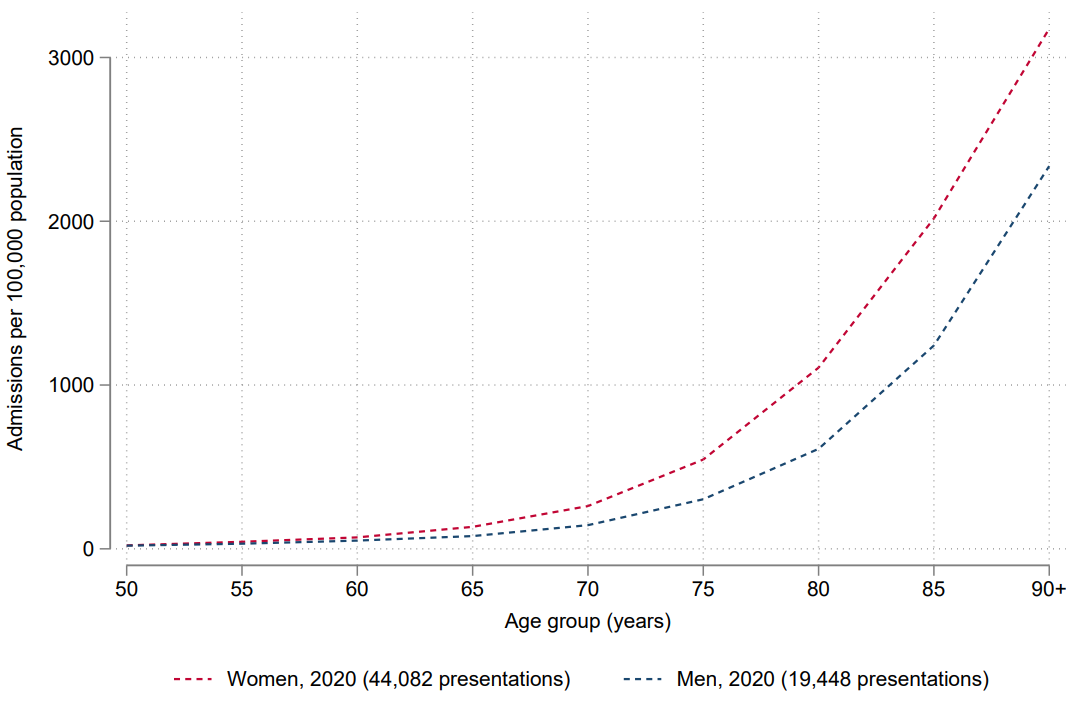


# eFigure 4: Hip fracture presentations and population estimates in adults age 50 years and older in England, January 2014 to October 2024.


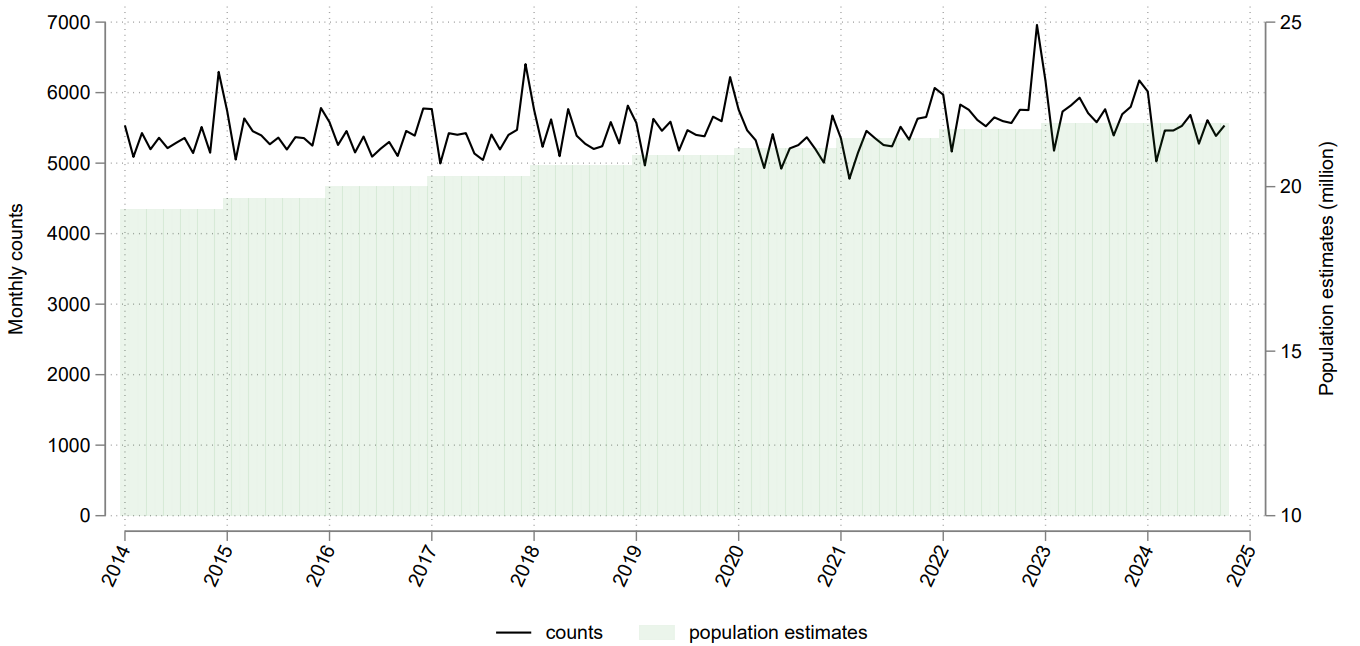


# eFigure 5: Age-sex-specific monthly hip fracture incidence rates in England, January 2014 to October 2024, in (a) women and (b) men aged 50 years and over.

(a) Women (b) Men


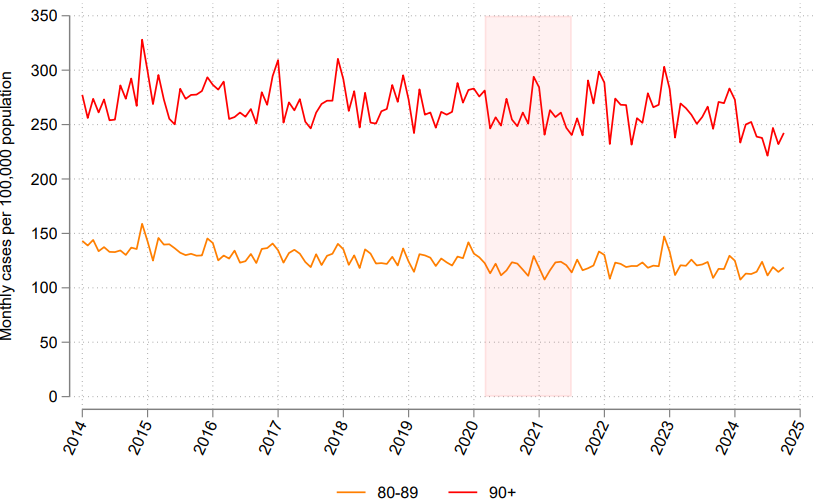

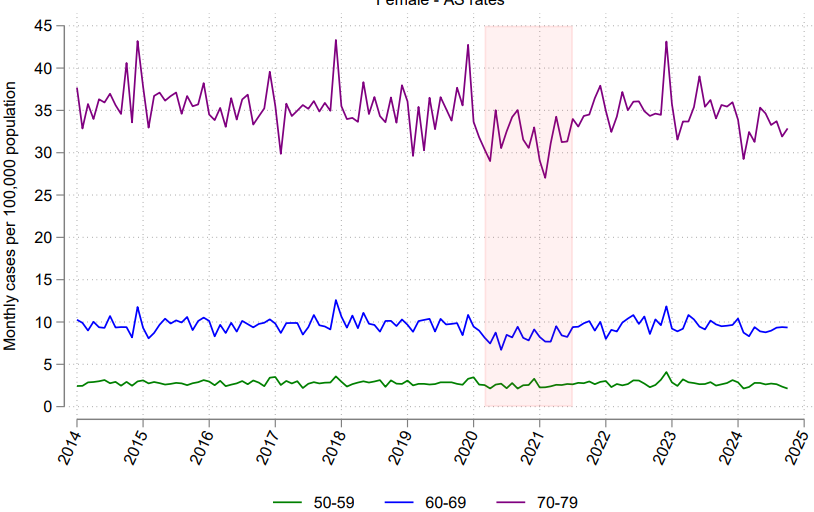

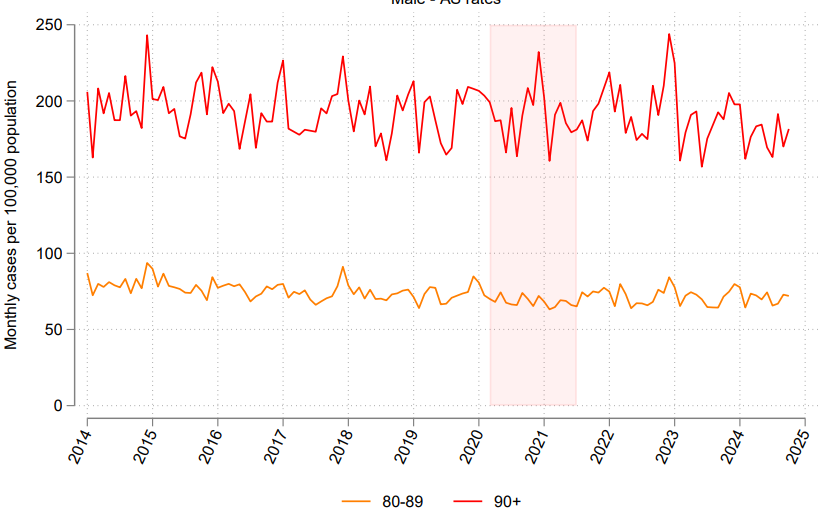

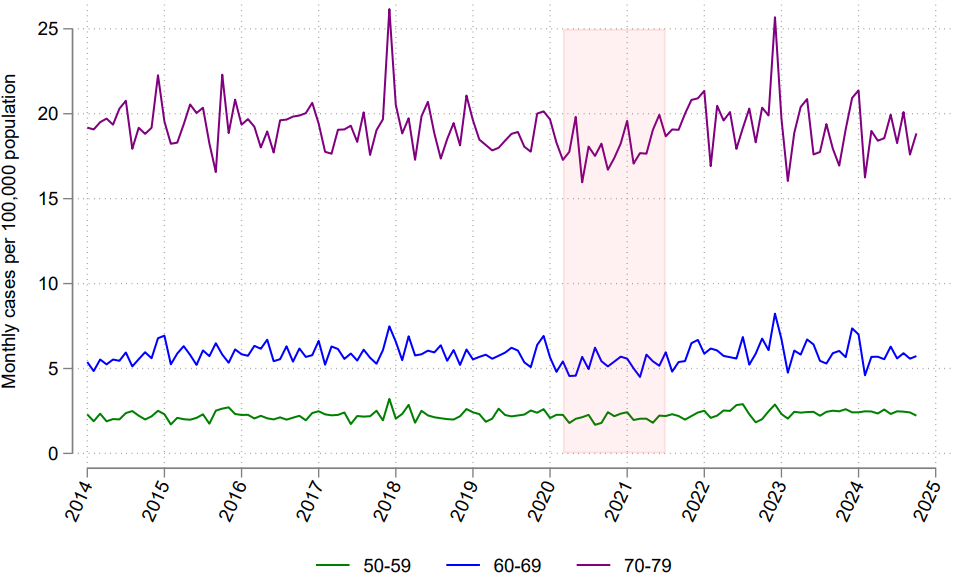


# eFigure 6: Age-sex-specific monthly hip fracture presentations in England, January 2014 to October 2024, in (a) women and (b) men aged 50 years and older.

a) Women (b) Men


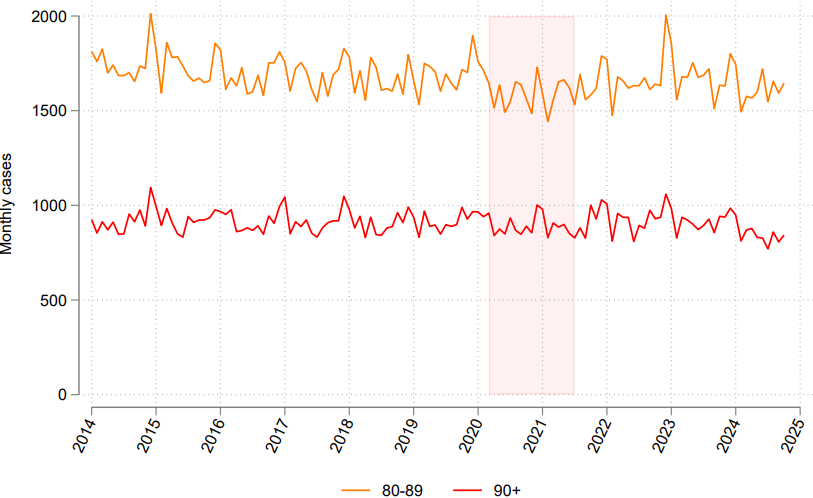

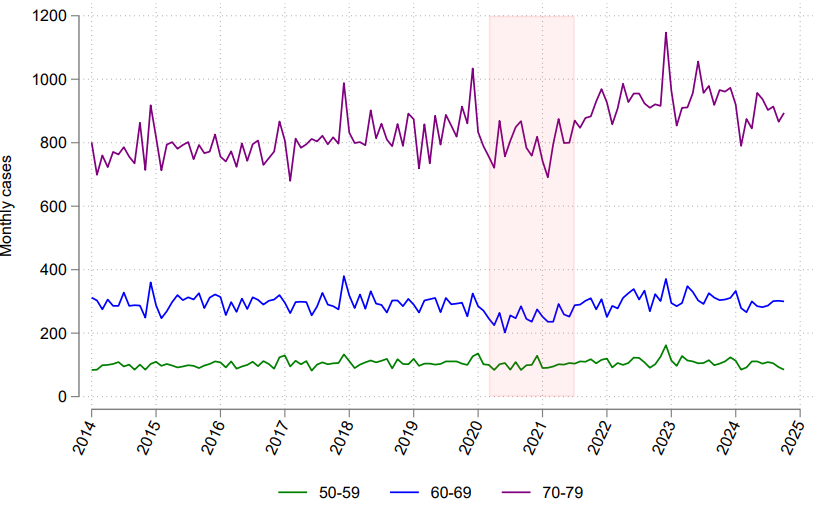

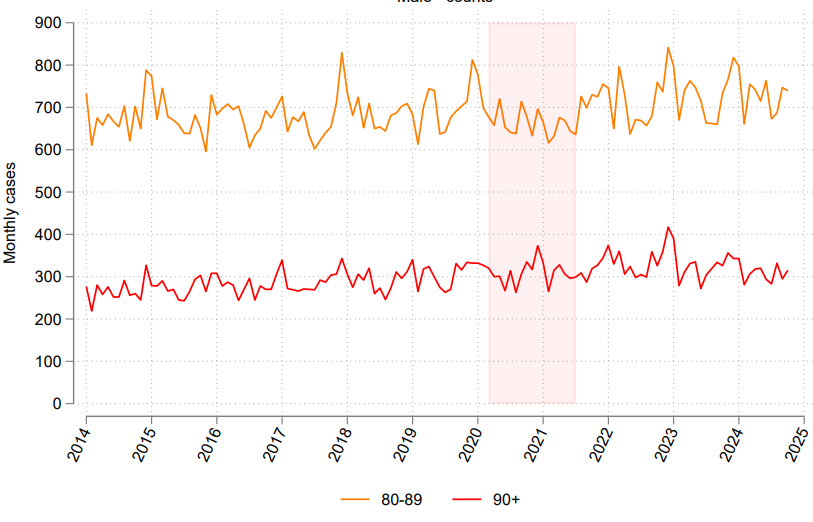

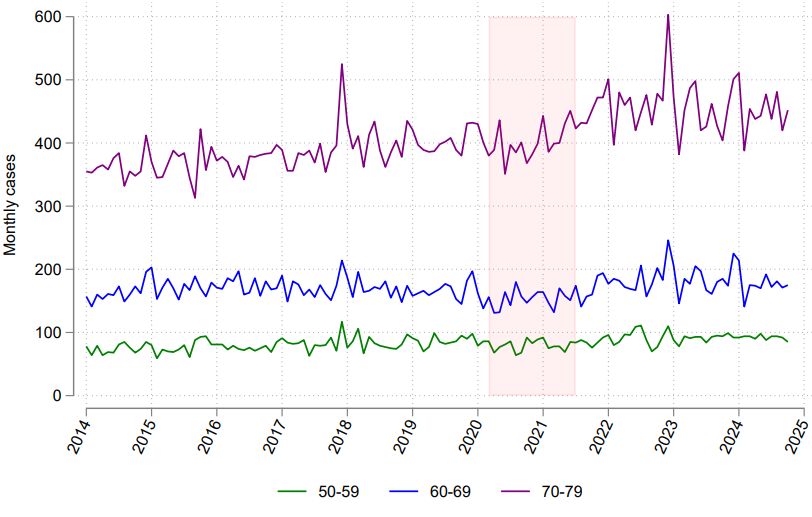


# eFigure 7: Age-sex-specific annual mid-year population estimates in England, January 2014 to October 2024, in (a) women and (b) men aged 50 years and older.

(a) Women (b) Men


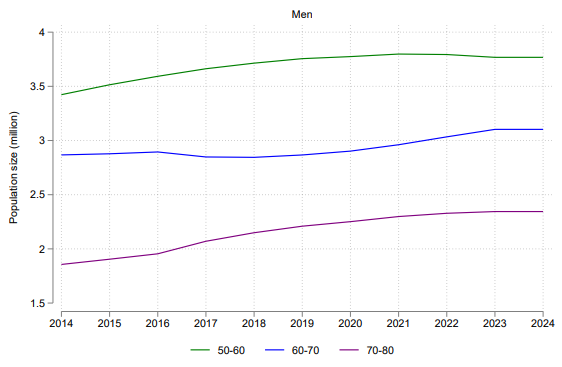

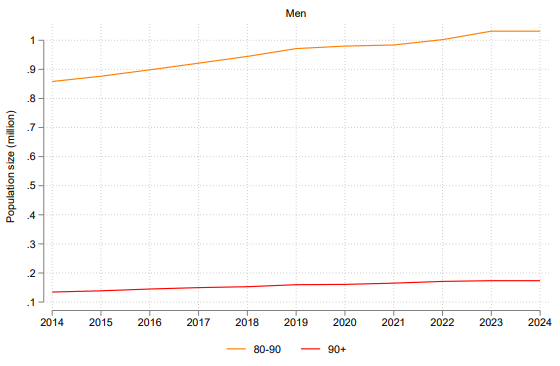

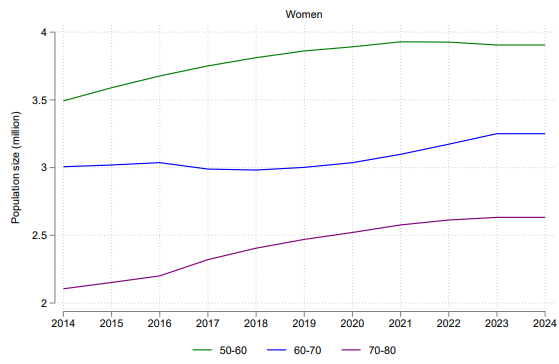

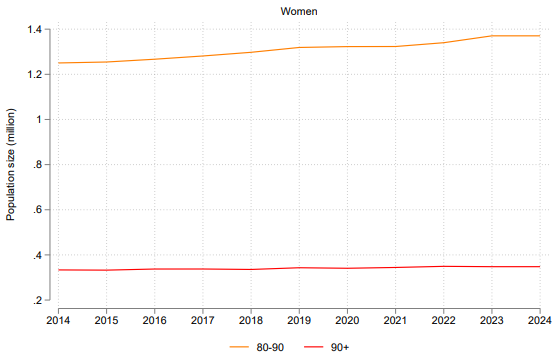


# eFigure 8: Monthly age-standardised hip fracture incidence rates in the most and least deprived quintiles in (a) men and (b) women aged 50 years and older in England, January 2014 to October 2024.

(a)


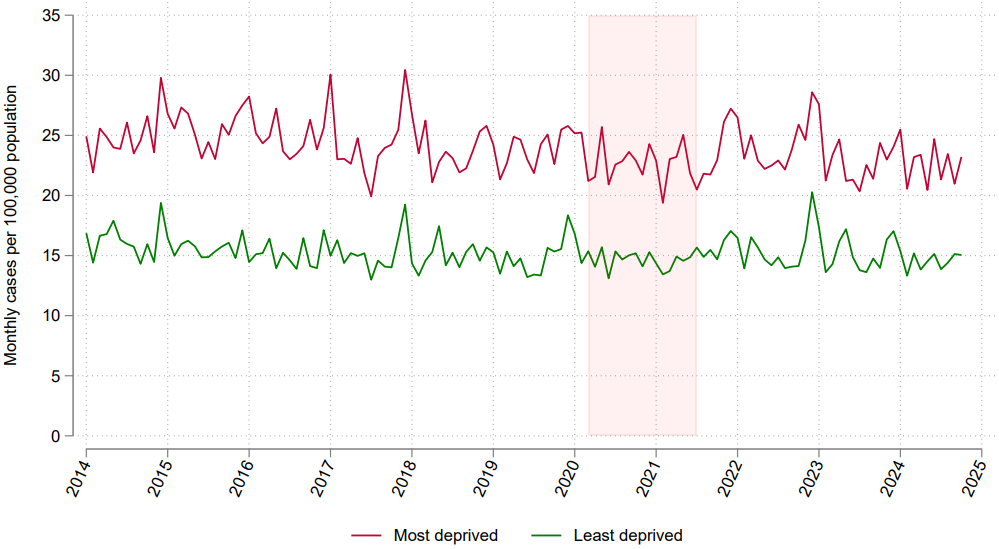


(b)


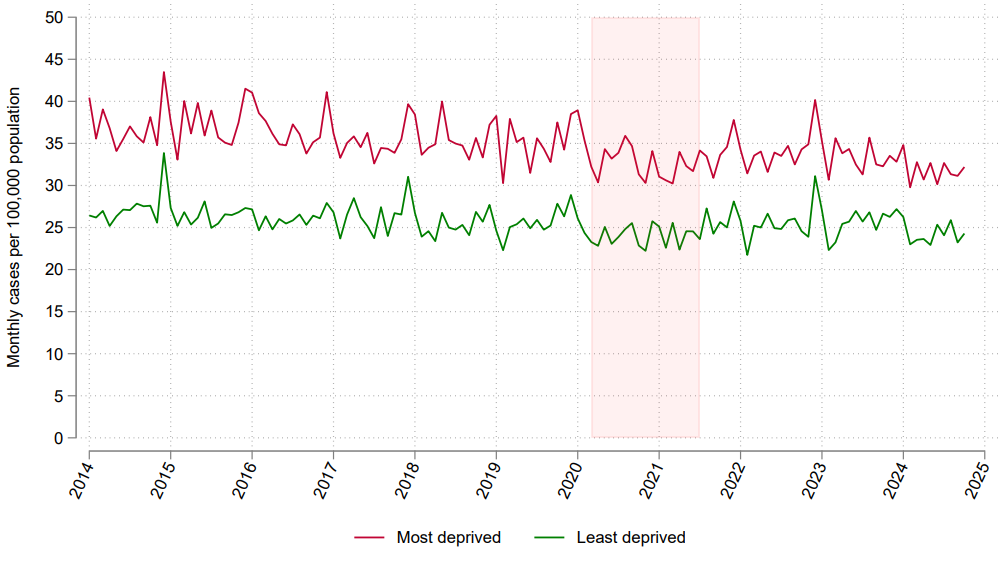

Supplement: Supplementary eFigs. 1–8 and eTables 1–5 [file mmc1.docx]
